# Supplementary material for: NOVA1 regulates hTERT splicing and cell growth in non-small cell lung cancer
Source: Nat Commun. 2018 Aug 6;9:3112. doi: 10.1038/s41467-018-05582-x (PMC6079032; doi:10.1038/s41467-018-05582-x)
Supplement: Supplementary file 1 — Supplementary Information [file 41467_2018_5582_MOESM1_ESM.pdf]

NOVA1 Regulates *hTERT* Splicing and Cell Growth in Non-Small Cell Lung Cancer

Ludlow et al. Supplementary information

Supplementary Table 1: Description of major *hTERT* splice isoforms

Isoform	Exon structure	Intron retention?	Biochemical function	References
**Full-length**	1-16.	no	Functional hTERT protein, maintains telomeres when in active telomerase holoenzyme (RNP)	[1, 2]
**Minus beta**	1-6, 9, and 10; PTC in 10.	no	Mostly degraded by non-sense mediated decay, some translated into protein and may play a role in DNA damage repair/ protection from apoptosis, may bind *hTERC (hTR)*	[3]
**Minus alpha**	1-16, alternative 3' splice acceptor site in exon 6 generates in frame shift of 36 nucleotides.	no	Dominant-negative, binds *hTERC (hTR)*	[1, 4]
**INS3**	1-16 plus, PTC in intron 14.	Retention of intron 14 nucleotide 623 to end on intron 14.	Dominant-negative, binds *hTERC (hTR)*	[5]
**INS4**	1-14, and alternative exon 16 3' splice site NT492, PTC in exon 14.	Retention of intron 14 nucleotides 1-600.	Dominant-negative, binds *hTERC (hTR)*	[5]
**DEL2**	1,3-16, PTC in exon 3.	No	Proposed mitochondrial *hTERT* variant, retains *hTERT* MLS in exon 1.	[6]

PTC – premature termination codon. RNP – ribonucleoprotein. NT- nucleotide.

Supplementary Table 2. Primers and probes				
Primer pairs	Primer name	Exon location	Primer sequence (5’-3’)	Probe – UPL or custom
1	hTERT 3' F	15/16 boundary	GGGTCACTCAGGACAGCCCAG	37
1	hTERT 3' R	16	GGGCGGGTGGCCATCAGT	
2	hTERT RT F	7/8 boundary	ACAGTTCGTGGCTCACCTG	52
2	hTERT RT R	8	GCGTAGGAAGACGTCGAAGA	
3	hTERT 5' F	2	AAGCATGCCAAGCTCTCG	17
3	hTERT 5' R	3	CAGGATCTCCTCACGCAGAC	
4	Minus Beta F	6/9 boundary	CAAGAGCCACGTCCTACGTC	58
4	Minus Beta R	9	CAAGAAATCATCCACCAAACG	
5	Minus Alpha F	5	GTTCAGCGTGCTCAACTACG	custom
5	Minus Alpha R	6	GTTCTGGGGTTTGATGATGC	
6	INS3 F	Retained intron 14	AGAGATGGAGCCACCCCGCA	custom
6	INS3 R	Retained intron 14 3’ end /exon 15 boundaries	AGCGACATCCCTGGGGGAAAAC	
7	INS4 F	Exon 14	TGAAAGCCAAGAACGCAGGTAT	custom
7	INS4 R	Intron 14 5’ end	TAAGCCCAGATTCACTCAGTCTCC	
8	MS2T1 F (assembly protein)	linear RNA (nt - 632)	GTCGCGGTAATTGGCGC	custom
8	MS2T1 R (assembly protein)	linear RNA (nt - 708)	GGCCACGTGTTTTGATCGA	
9	MS2T2 F (lysis protein)	linear RNA (nt - 1693)	CCTCAGCAATCGCAGCAAA	custom
9	MS2T2 R (lysis protein)	linear RNA (nt - 1807)	GGAAGATCAATACATAAAGAGTTGAACTTC	
10	TERT CLIP intron 5 F		GTCAGAGAAGGAACCGCAAC	Evagreen
10	TERT CLIP intron 5 R		TGAGCCTCAGGACAGGAGAC	Evagreen
11	TERT CLIP exon 6 F		GCATCATCAAACCCCAGAAC	Evagreen
11	TERT CLIP exon 6 R		CTTCTGGACCACGGCATAC	Evagreen
12	TERT CLIP intron 6 F		CATGTTCATGCTGTGTGCTG	Evagreen
12	TERT CLIP intron 6 R		AGGAAGGAGTAAGGCCAAGG	Evagreen
13	TERT CLIP intron 6.2 F		CAGTCTTTACTGTGTCAGCTTGC	Evagreen
13	TERT CLIP intron 6.2 R		AACCAATCCCACCTACCC	Evagreen
14	TERT CLIP intron 6.3 F		CCTGTAGTGGGTCTGCAGGT	Evagreen
14	TERT CLIP intron 6.3 R		CCCGACCATAGTTCAAAGGA	Evagreen
15	TERT CLIP intron 6.4 F		CTGCAATCCCTCCAGCAC	Evagreen
15	TERT CLIP intron 6.4 R		CACAGTCACGTGGCACAAG	Evagreen

16	TERT CLIP intron 7 F		GCCTAAGCCCATGTGTGTCT	Evagreen
16	TERT CLIP intron 7 R		TTTGGGGTCAGTGTTTGTGA	Evagreen
17	TERT CLIP exon 8 F		CTGAATGAGGCCAGCAGTG	Evagreen
17	TERT CLIP exon 8 R		GGCACATGAAGCGTAGGAAG	Evagreen
18	TERT CLIP intron 8 F1		TGGCAGCAGAGTGAATTTTG	Evagreen
18	TERT CLIP intron 8 R1		TCTGGGGAGGTGACTTTGTC	Evagreen
19	TERT CLIP intron 8 F2		GCGCCTGGCTAATTTTTGTA	Evagreen
19	TERT CLIP intron 8 R2		CCTGAGGTCAGGAGTTCGAG	Evagreen
20	TERT CLIP exon 9 F		ATCCTCTCCACGCTGCTCT	Evagreen
20	TERT CLIP exon 9 R		ACAGCTTGTTCTCCATGTCG	Evagreen
21	TERT CLIP intron 9 F		CAGCCCCTTCTTGGTATGAA	Evagreen
21	TERT CLIP intron 9 R		GTGCTTCCTCGGTGTTGAAT	Evagreen
22	GLRA CLIP region 1 F		CAAATGGCTCCTGGGTAAAA	Evagreen
22	GLRA CLIP region 1 R		CCTTCAGCCAAACTACAGTGC	Evagreen
23	GLRA CLIP region 2 F		GGCCTTCTCATCATTTTTGG	Evagreen
23	GLRA CLIP region 2 R		ACAGGGTCTGCTTCTTGGAA	Evagreen
24	GLRA CLIP region 3 F		CCTGCCTTTCCAACAGCTTA	Evagreen
24	GLRA CLIP region 3 R		CACGTAGGAAAGGTTGGGATT	Evagreen
25	GAPDH CLIP F		AGCCACATCGCTCAGACAC	Evagreen
25	GAPDH CLIP R		GCCCAATACGACCAAATCC	Evagreen
26	TS	ddTRAP	AATCCGTCGAGCAGAGTT	Evagreen
26	ACX	ddTRAP	GCGCGGCTTACCCTTACCCTTACCCTAACC	Evagreen
27	hTERT DR8 YAAY mutant F	CRIPSR genotyping	GGCTTGAGTGCAGTGGCGCG	Gel based
27	hTERT DR8 YAAY mutant R	CRIPSR genotyping	TTTCGGGAAGCGCTATAG	Gel based
28	hTERT DR8 F deletion	CRIPSR genotyping	ACCTCCCCAGAGAAGCCACCAC	Gel based
28	hTERT DR8 R deletion	CRIPSR genotyping	TGCACACAGAAGGCATGGC	Gel based
29	hTERT DR8 F deletion 2	CRIPSR genotyping	TGGCAGCAGAGTGAATTTTG	Gel based
29	hTERT DR8 R deletion 2	CRIPSR genotyping	TCAGCATGACCAACGAGAAG	Gel based
30	hTERT 5-9 F 2109	hTERT RT-PCR	GCCTGAGCTGTACTTTGTCAA	Gel based
30	hTERT 5-9 R 2531R	hTERT RT-PCR	AGGCTGCAGAGCAGCGTGGAGAGG	Gel based
Notes: UPL – Universal Probe Library (Roche).				

Notes: Custom probe sequence available on request.

Supplementary Figure 1

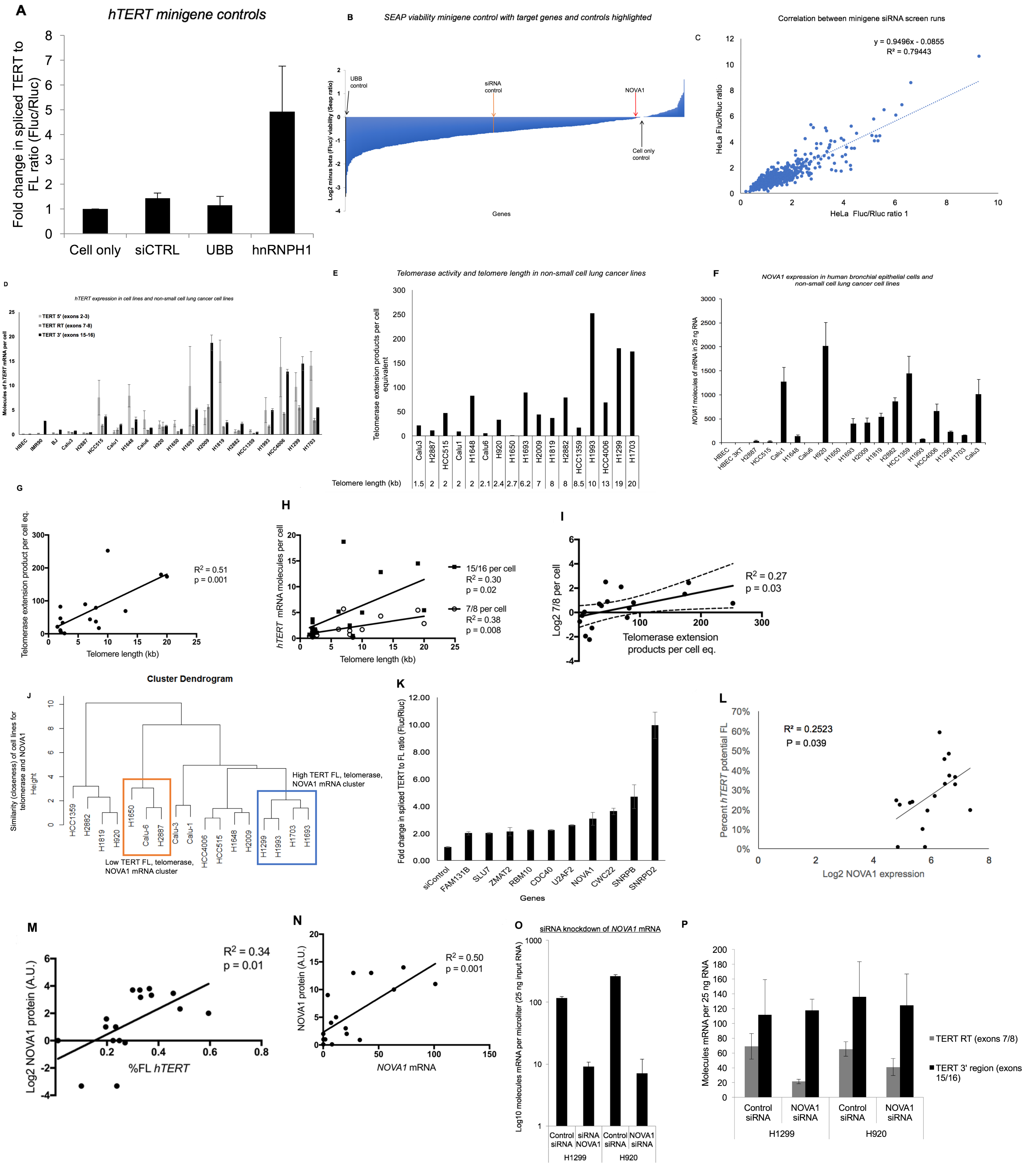

Supplementary Figure 1

(A) *hTERT* minigene luciferase assay of controls used in the screen. 'Cell only' contains cells and media. 'siCTRL' is the siRNA scrambled control. 'UBB' is a pool of four siRNAs targeting ubiquitin. hnRNPH1 is a pool of four siRNAs targeting the splicing factor hnRNPH1. Data are expressed as means ± standard deviation.

(B) Secreted embryonic alkaline phosphatase (SEAP) viability assay. Viability for the siRNA scrambled control (siRNA control), 'cell only' control, *NOVA1*, and UBB siRNA pools are pointed out. All data are expressed relative to cell only control, set at a value of '1'. 'Cell only' contains cells and media. 'siCTRL' is the siRNA scrambled control. 'UBB' is a pool of four siRNAs targeting ubiquitin.

(C) Pearson's r correlation between minigene siRNA screen repeats.

(D) Steady state expression of *hTERT* mRNAs across the *hTERT* locus showing general expression patterns in cell lines with and without telomerase activity. Expression is expressed in molecules per cell. Each data point represents three individual reverse transcription reactions repeated twice in droplet digital PCR. Data are expressed at means ± standard errors of the mean.

(E) Telomerase enzyme activity as determined by droplet digital TRAP (ddTRAP) and telomere length (terminal restriction fragment (TRF) lengths from Frink et al. 2016, Oncotarget). Telomerase was determined using 50 cell equivalents input.

(F) *NOVA1* mRNA levels in normal and transformed cells. Data are expressed per 25 ng of cDNA input. Data are expressed as means ± standard errors of the mean.

(G) Pearson's r correlation between telomere length (TRF) and ddTRAP determined telomerase enzyme activity analysis. Telomerase determined by ddTRAP (above in E) using 50 cell equivalents (eq.).

(H) Pearson's r correlation between telomere length (TRF) and mRNA expression of *hTERT* RT domain (exons 7 and 8) and 3' end (exons 15 and 16) on a per cell basis.

(I) Pearson's r correlation between telomerase enzyme activity (ddTRAP, 50 cell equivalents) and log2 mRNA expression of *hTERT* RT domain (exons 7 and 8) on a per cell basis.

(J) Cluster dendrogram for "network discovery analysis". Cluster analysis shows the 'closeness' of the cell lines for *hTERT* mRNA expression, telomerase activity and *NOVA1* mRNA expression.

(K) Minigene targets (2-fold or greater) that overlapped with data generated from the microarray analyses (both "gene discovery" and "network" overlapping genes are included). Data are expressed as means ± standard errors of the mean.

(L) Pearson's r correlation analysis of log2 *NOVA1* mRNA expression with percent *hTERT* potential full-length (FL) splicing (mRNA).

(M) Pearson's r correlation analysis of percent full-length (FL) *hTERT* with log2 *NOVA1* protein expression.

(N) Pearson's r correlation between *NOVA1* protein levels and mRNA levels.

(O) Validation of *NOVA1* siRNA mediated knockdown at the mRNA level in H1299 and H920 cells. Cells were collected and analyzed 72 hours after siRNA transfection.

(P) mRNA expression of *hTERT* following siRNA mediated knockdown of *NOVA1* in H1299 and H920 cells (as in O). Shows reduction of RT domain containing transcripts (exons 7 and 8) and maintenance of total levels of *hTERT* expression (3' exons 15 and 16). Data are expressed as means ± standard errors of the mean.

TRF – terminal restriction fragment length for determination of telomere length.

Supplementary Figure 2

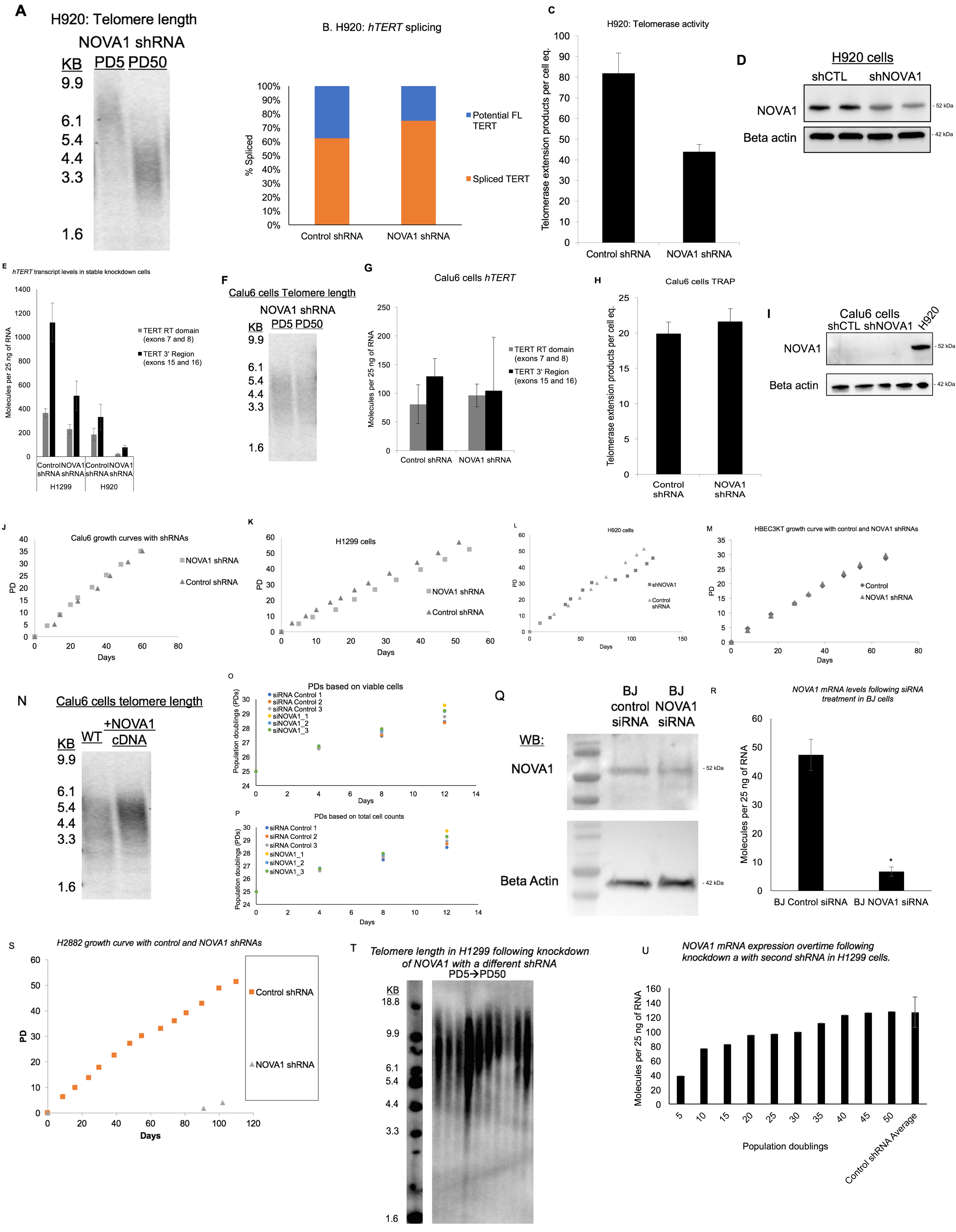

Supplementary Figure 2

(A) Terminal Restriction Fragment (TRF) analysis of H920 cells with control non-silencing shRNA or *NOVA1* shRNA mediated knockdown at population doubling (PD) 5 and 50.
(B) *hTERT* RT-ddPCR splicing isoform analysis of H920 cells with control non-silencing shRNA or *NOVA1* shRNA mediated knockdown over the course of the experiment.
(C) Telomerase activity determined by ddTRAP in H920 cells with control non-silencing shRNA or *NOVA1* shRNA mediated knockdown over the course of the experiment. 50 cell equivalents (eq.) were used for the ddTRAP assays (n = 6). Data are expressed as means ± standard errors of the mean.
(D) Western blot showing *NOVA1* protein levels in H920 cells with control non-silencing shRNA or *NOVA1* shRNA mediated knockdown. Representative images shown (n = 2).
(E) H1299 and H920 steady state *hTERT* transcript levels in control non-silencing shRNA or stable shRNA knockdown of *NOVA1* cells. RT-ddPCR was used to assay *hTERT* at two different regions of the gene to infer expression of RT domain (exons 7 and 8) compared to total transcript expression levels (exons 15 and 16; n = 6).
(F) Terminal Restriction Fragment (TRF) analysis of Calu6 cells with control non-silencing shRNA or *NOVA1* shRNA mediated knockdown at population doubling (PD) 5 and 50. Calu6 cells lack *NOVA1* protein (see Supplementary Figure 2I).
(G) Calu6 cells steady state *hTERT* transcript levels in stable control non-silencing shRNA or shRNA knockdown of *NOVA1*. Calu6 cells lack *NOVA1* protein (see Supplementary Figure 2I). Data are expressed as means ± standard errors of the mean.
(H) Telomerase activity determined by ddTRAP in Calu6 cells with control non-silencing shRNA or *NOVA1* shRNA mediated knockdown over the course of the experiment. Calu6 cells lack *NOVA1* protein. 50 cell equivalents (eq.) were used in the assay. Data are expressed as means ± standard errors of the mean.
(I) Western blot of Calu6 cells with control non-silencing shRNA (CTL) or shRNA to *NOVA1*. H920 cells protein lysate was used as a positive control for NOVA1 antibody.
(J) Calu6 cells growth curves with control non-silencing shRNA and *NOVA1* shRNA.
(K) H1299 cells growth curves with control non-silencing shRNA and *NOVA1* shRNA.
(L) H920 cells growth curves with control non-silencing shRNA and *NOVA1* shRNA.
(M) HBEC3KT growth curve with control non-silencing shRNA and *NOVA1* shRNA.
(N) Wild type (WT) Calu6 cells and Calu6 cells with *NOVA1* expression (lentiviral V-5 tagged *NOVA1* stable cell line) terminal restriction fragment length analysis (TRF).
(O and P) Growth curves of BJ cells treated with control non-silencing or *NOVA1* siRNA. Population doublings based on viable cells (O) or total cell number (P) are shown).
(Q) Western blot of *NOVA1* in siRNA treated BJ cells. Representative image (n = 2).
(R) RT-ddPCR analysis of *NOVA1* mRNA levels following siRNA treatment in BJ cells. Three replicates in each condition. Data are expressed as means ± standard errors of the mean. Student's t test set at $p < .05$ for significance, *).
(S) H2882 growth curve with *NOVA1* or control non-silencing shRNAs.
(T) Terminal restriction fragment length analysis (TRF) in H1299 cells with a second shRNA to *NOVA1*. Ten samples in successive passage are shown from 5 PDs to 50 PD, every 5 PDs.
(U) RT-ddPCR of *NOVA1* mRNA levels in the same ten samples in panel T. Shows loss of *NOVA1* KD via a selective growth advantage of cells with control levels of *NOVA1*. Data are expressed as means ± standard errors of the mean.
RT = reverse transcription; ddPCR = droplet digital PCR; PD = population doublings

Supplementary Figure 3

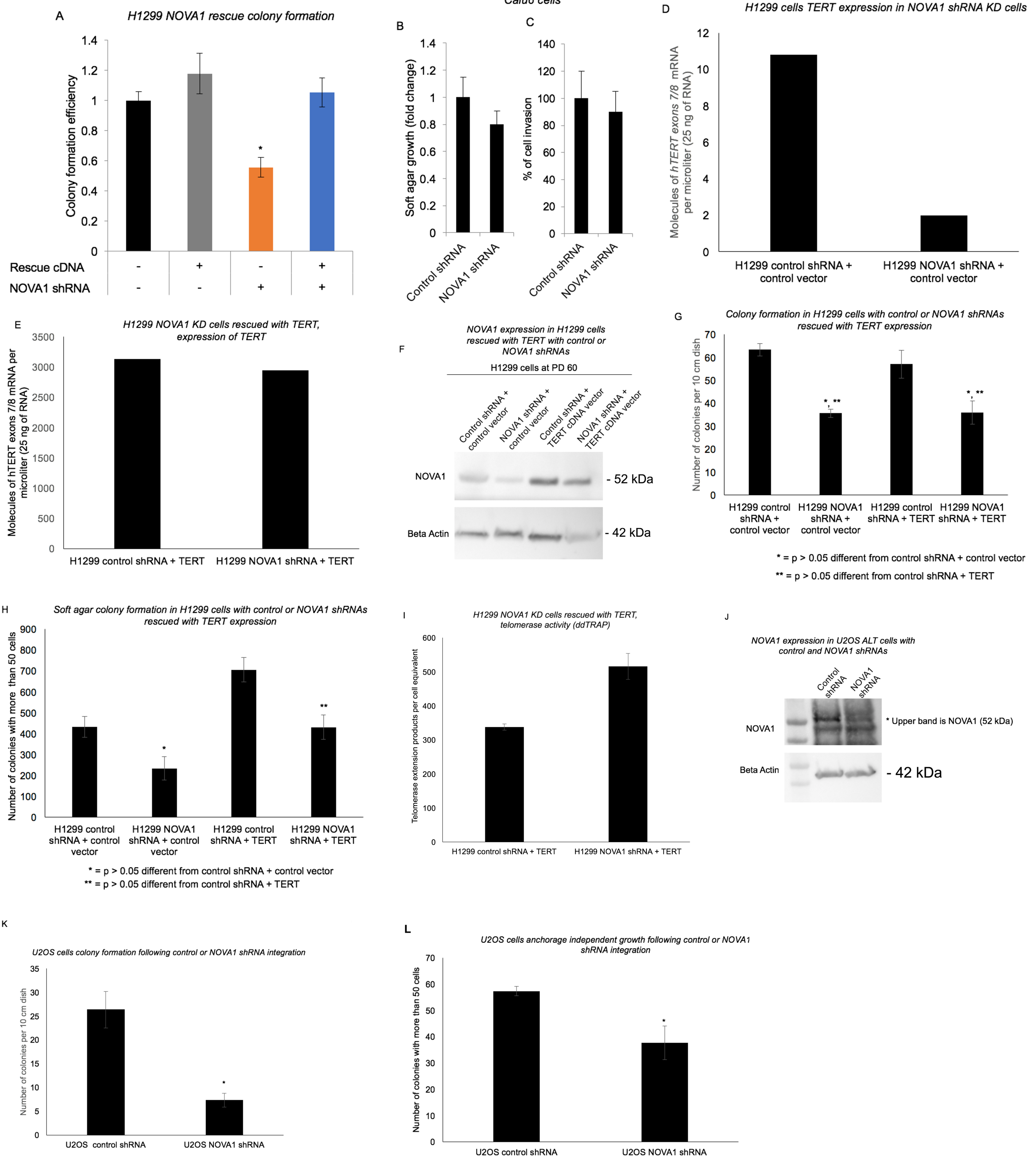

Supplementary Figure 3

(A) Colony formation assay in H1299 rescue series cells. Analysis was performed in biological triplicate and technical duplicate (statistical significance tested with Student's *t* test, $p > 0.05$).

(B-C) Tumorigenicity analysis in Calu6 cells with control and NOVA 1 shRNAs. Growth on soft agar and invasion through matrigel (Boyden Chamber) analysis was performed in biological triplicate and technical duplicate (statistical significance tested with Student's *t* test, $p > 0.05$).

(D) Expression of endogenous hTERT expression at exons 7/8 in H1299 shRNA control or shRNA NOVA1. Single sample as confirmation.

(E) Expression of ectopic hTERT expression at exons 7/8 in H1299 shRNA control or shRNA NOVA1. Single sample as confirmation.

(F) Western blot of NOVA1 in H1299 shRNA controls or NOVA1 with or without hTERT expression vector. Single sample analyzed as confirmation.

(G) Colony formation assay in H1299 shRNA cells with or with ectopic hTERT expression. Analysis was performed in triplicate (three biological replicates of each condition; statistical significance tested with Student's *t* test, $p > 0.05$).

(H) Soft agar assay in H1299 shRNA cells with or with ectopic hTERT expression. Analysis was performed in triplicate (three biological replicates of each condition; statistical significance tested with Student's *t* test, $p > 0.05$).

(I) NOVA1 expression by western blot. Representative sample. Single samples for control and NOVA1 shRNAs.

(J) Colony formation assay in U2OS shRNA cells. Analysis was performed in triplicate (three biological replicates of each condition; statistical significance tested with Student's *t* test, $p > 0.05$).

(K) Soft agar assay in U2OS shRNA cells. Analysis was performed in triplicate (three biological replicates of each condition; statistical significance tested with Student's *t* test, $p > 0.05$).

Supplementary Figure 4

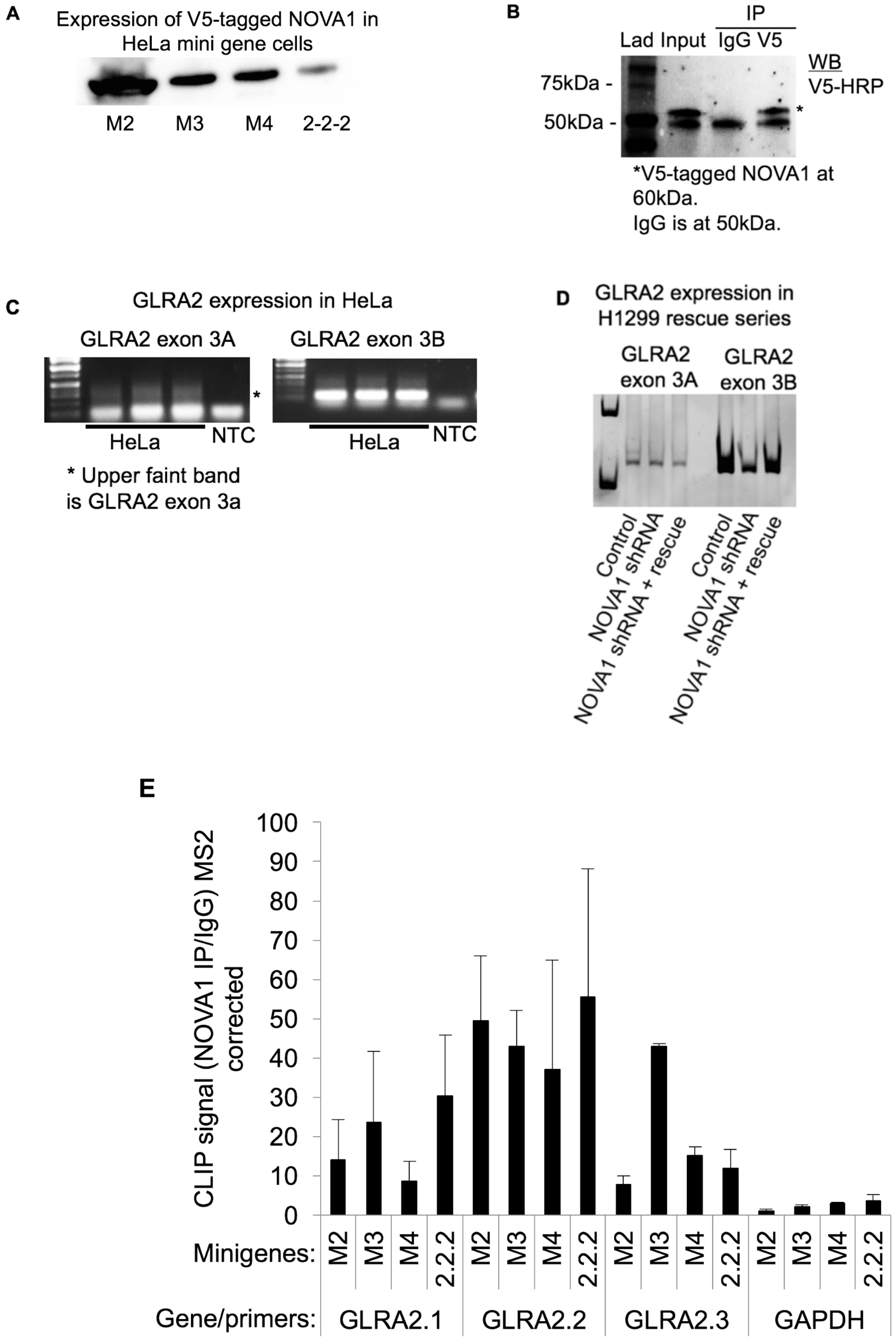

Supplementary Figure 4
(A) Expression of V5-Tagged NOVA 1 in HeLa cells containing *hTERT* minigenes M2, M3, M4, and 2-2-2. Single confirmatory blot.
(B) Representative immunoprecipitation of V5-tagged NOVA 1 from HeLa cells with *hTERT* minigenes. Single confirmatory blot.
(C) *GLRA2* mRNA expression of alternatively spliced exons 3A and 3B in HeLa cells with *hTERT* minigenes (n = 3).
(D) *GLRA2* mRNA expression of alternatively spliced exons 3A and 3B in H1299 cells with shRNA control, shRNA against NOVA 1, or with shRNA against NOVA 1 rescued with a NOVA 1 cDNA resistant to NOVA 1 shRNA. Representative image shown (n=3).
(E) Compiled CLIP data showing successful pulldown of NOVA 1 target gene *GLRA2* and a negative control RNA, *GAPDH* (n = 3 independent pull downs).

Supplementary Figure 5

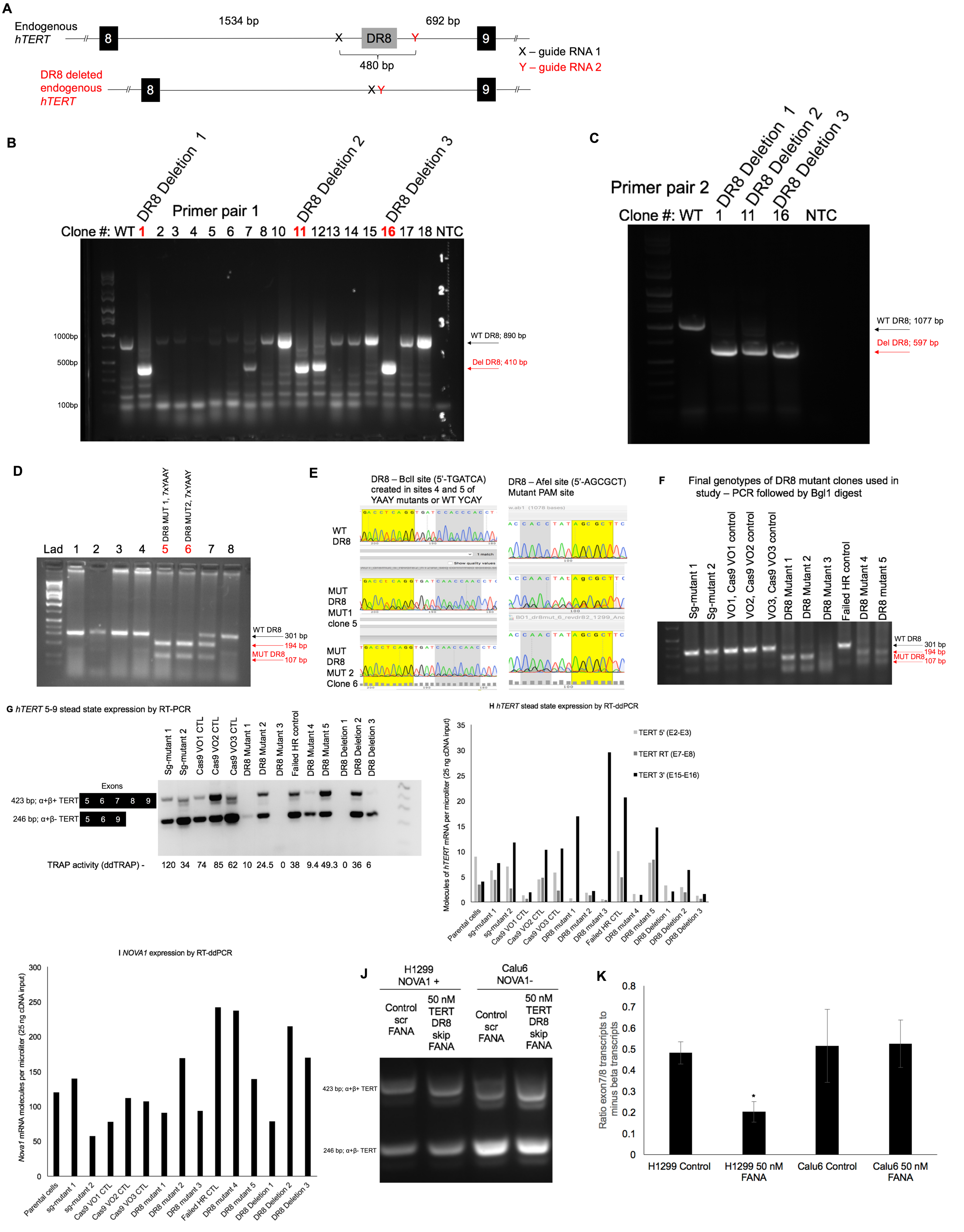

Supplementary Figure 5

(A) Cartoon of hTERT locus showing DR8 in intron 8 deletion scheme and primer locations for genotyping.

(B) Agarose gel following PCR with primer pair 1 to detect hTERT DR8 deletion.

(C) Agarose gel following PCR with primer pair 2 in WT and mutants 1 (DR8 Deletion 1), 11 (DR8 Deletion 2), and 16 (DR8 Deletion 3).

(D) Agarose gel of digested PCR product of WT (lane 1) and attempted CRISPR/Cas9 manipulation of intron 8 to be NOVA1 resistant.

(E) Sanger sequencing confirming gel analysis of manipulated alleles.

(F) Agarose gel of showing genotypes of final hTERT DR8 mutant and WT clones used in this study.

(G) *hTERT* 5-9 gel PCR analysis of steady state mRNA levels of mutant H1299 cells. TRAP quantification (telomerase extension products per cells equivalent) shown below each clone's lane.

(H) *hTERT* ddPCR analysis of steady state mRNA levels in mutant H1299 cells.

(I) *NOVA1* mRNA expression (exon 4 containing) levels in mutant H1299 cells.

(J) H1299 and Calu6 cells expression of *hTERT* (RT-PCR) exon 5-9 following 48 hrs. treatment with 2'FANA antisense (50 nM) *hTERT* DR8+19 oligos. Representative image.

(G) Quantification of *hTERT* splicing following treatment with 2'FANA antisense (50 nM) *hTERT* DR8+19 oligos (three biological replicates of each condition; statistical significance tested with Student's *t* test, $p > 0.05$).

Supplementary Figure 6. Primer location for measurement of hTERT mRNA expression and splicing variants

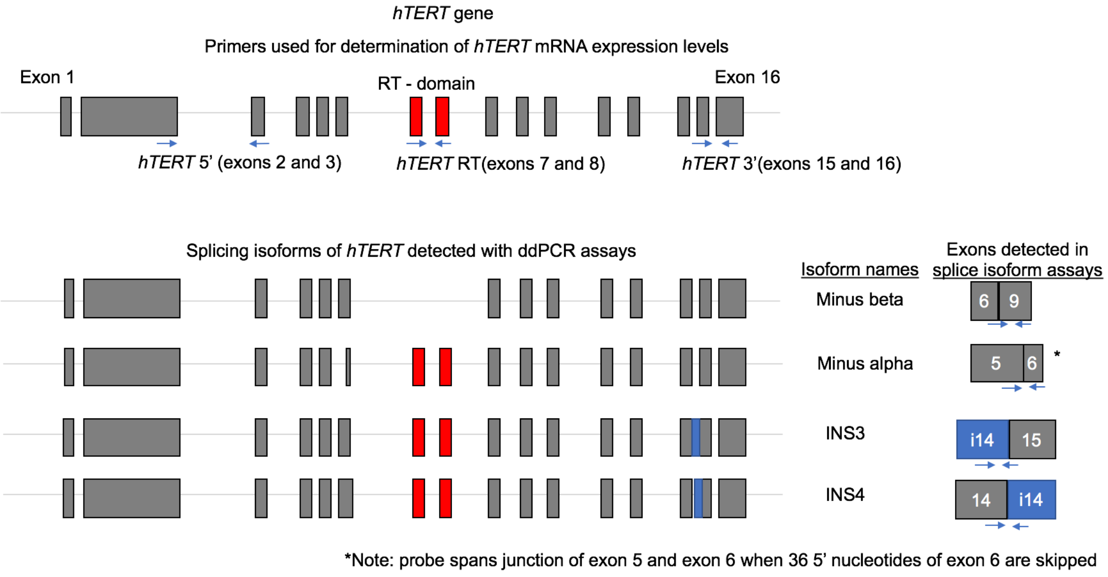

Supplementary Figure 6. hTERT primer locations. Cartoon schematic showing locations of selected primers and probes for measurement of hTERT splicing and steady state mRNAs. Upper cartoon shows primer locations used to measure hTERT steady state mRNAs at three different locations across the gene locus. Lower cartoons show intronic and exonic structures of 4 splice variants and the location of the primers used to measure these variants in this manuscript.

Supplementary Figure 7 – Uncropped blots and gel images (page 1 of 3)

Figure 1E

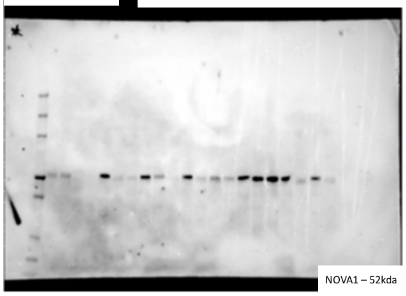

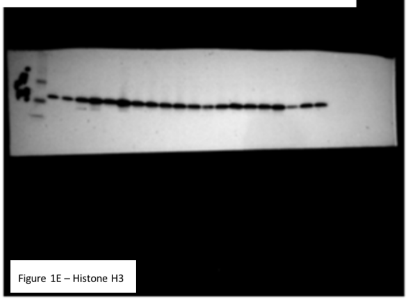

Figure 2A

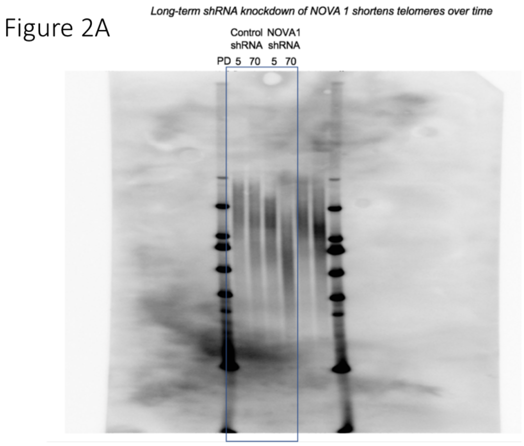

Figure 2C

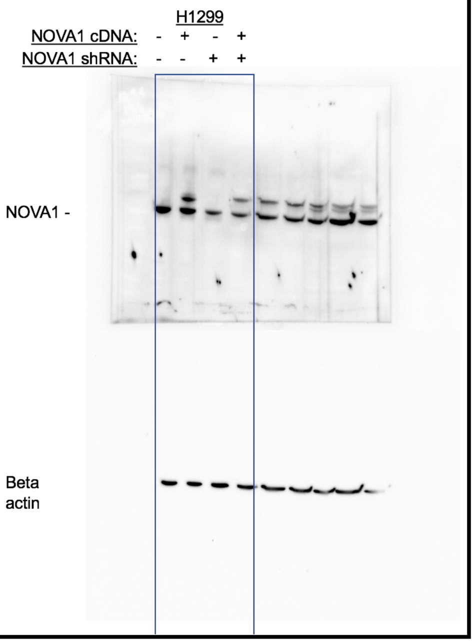

Figure 2D hTERT 5-9 splicing in rescue series

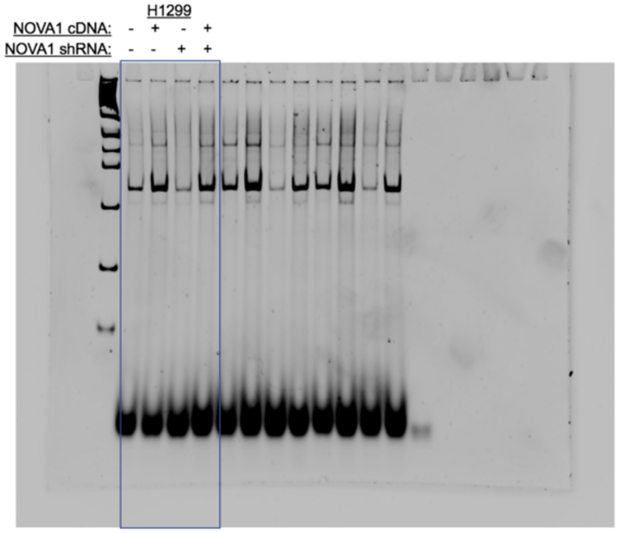

Figure 2F NOVA1 over expression in calu6

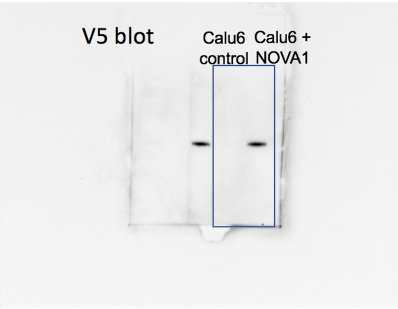

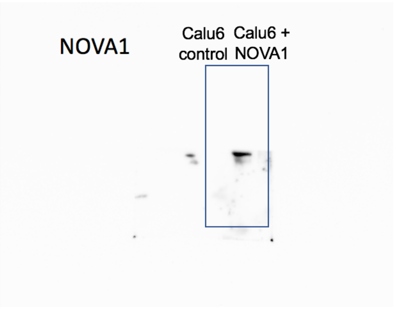

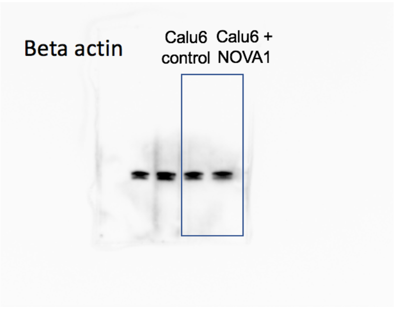

Supplementary Figure 2

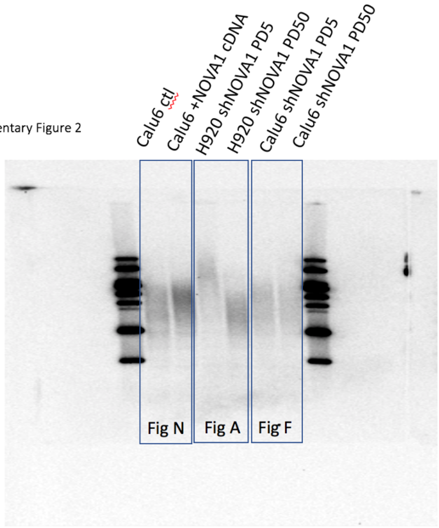

Supplementary Figure 2 D – NOVA1 western H920 cells

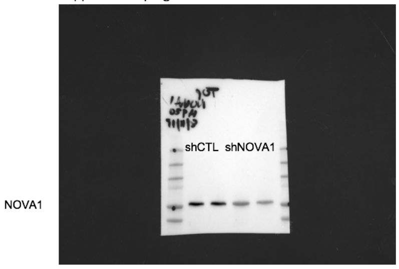

Supplementary Figure 2 D – NOVA1 western H920 cells

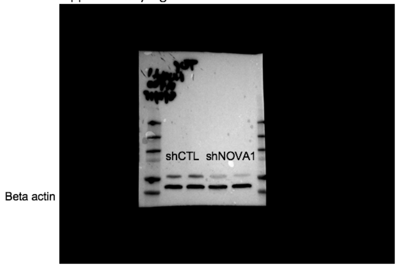

Supplementary Figure 2 – NOVA1 western Calu6 cells

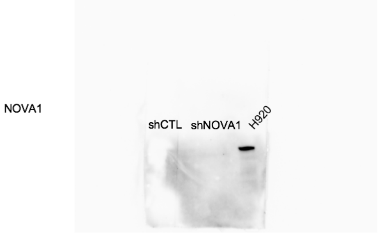

Supplementary Figure 2 – NOVA1 western Calu6 cells

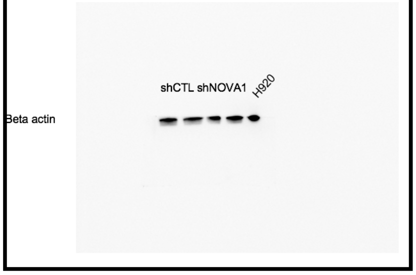

Supplementary Figure 7 – Uncropped blots and gel images (page 2 of 3)

Supplementary Figure 2
Western blot NOVA1 and beta actin BJ siRNA

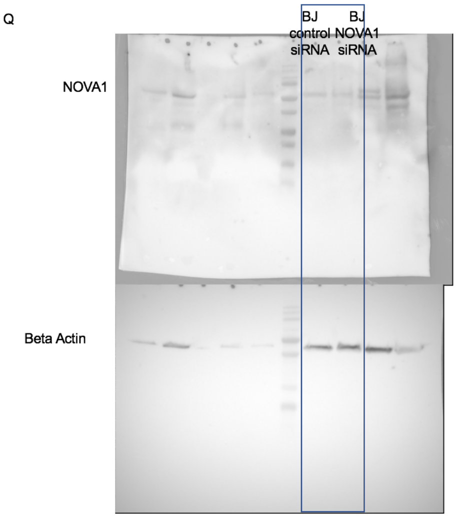

Supplementary Figure 2 T – TRF in H1299 with NOVA1 shRNA #2

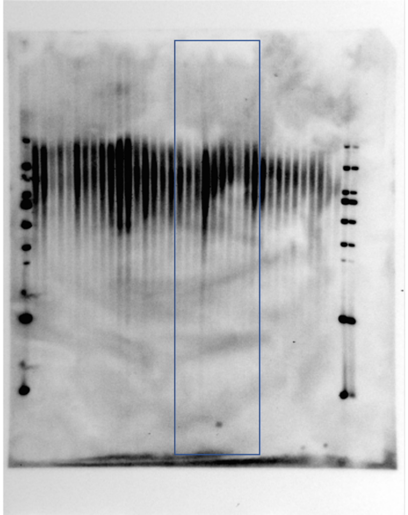

Supplementary Figure 3F uncropped gels

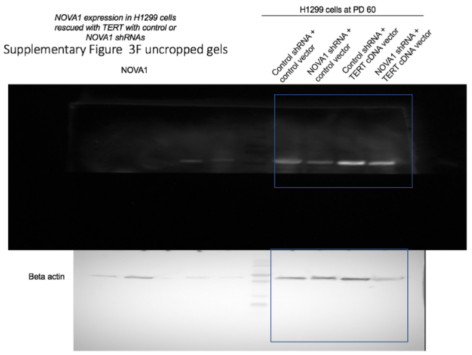

Supplementary Figure 3J uncropped gels

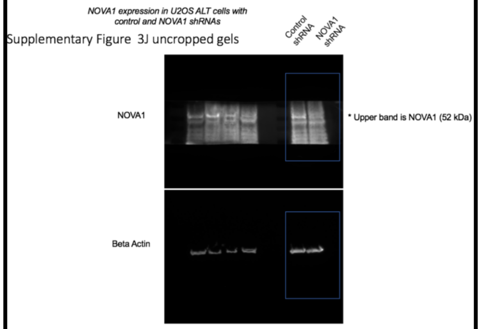

Supplementary Figure 4A

- V5 expression in Hela minigene cells

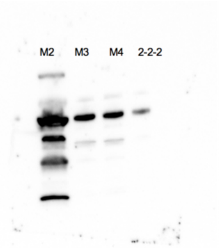

Supplementary figure 4B

- V5 IP in CLIP

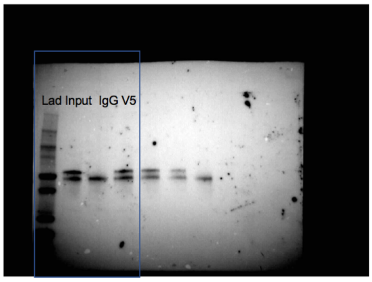

Supplementary Figure 4C

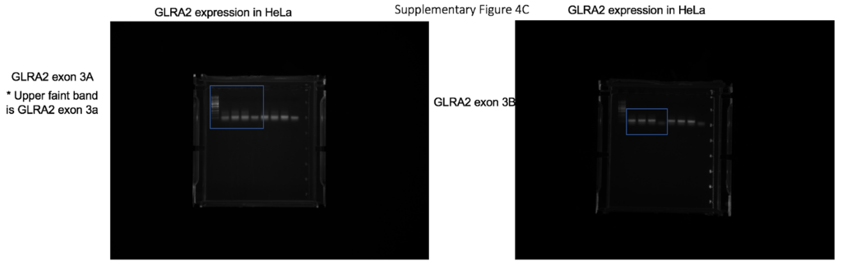

Supplementary Figure 4D

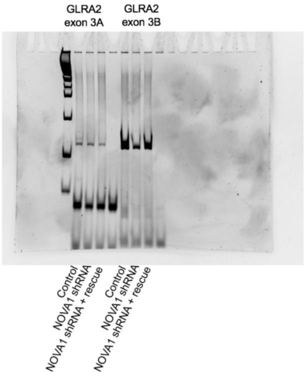

Figure 4F NOVA1 pull down 1kb blot

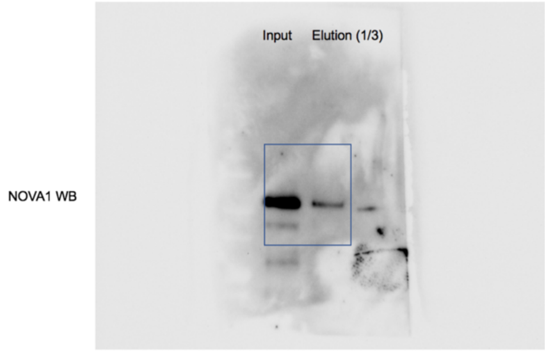

Figure 4H NOVA1 DR8 RNA pull down uncropped blot

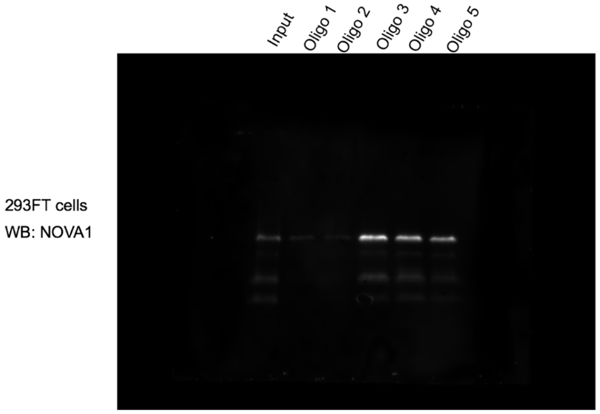

Supplementary Figure 7 – Uncropped blots and gel images (page 3 of 3)

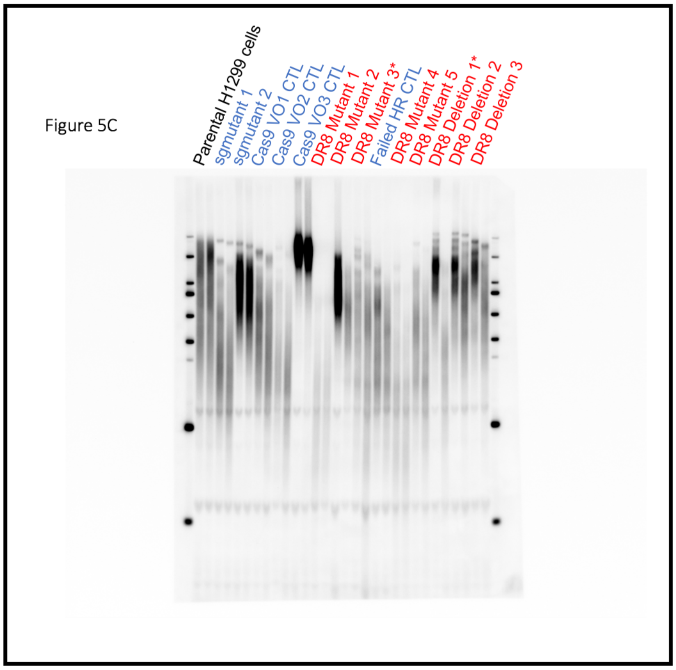

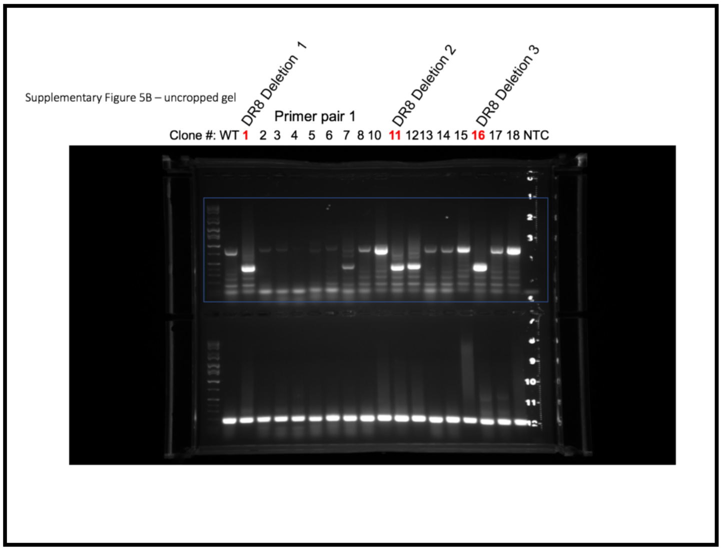

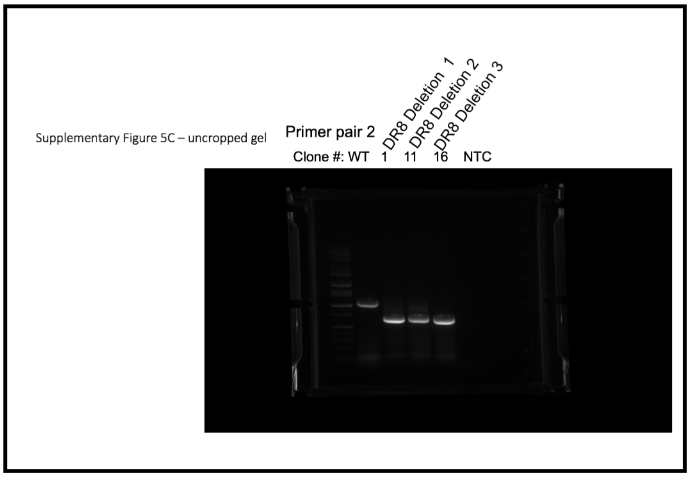

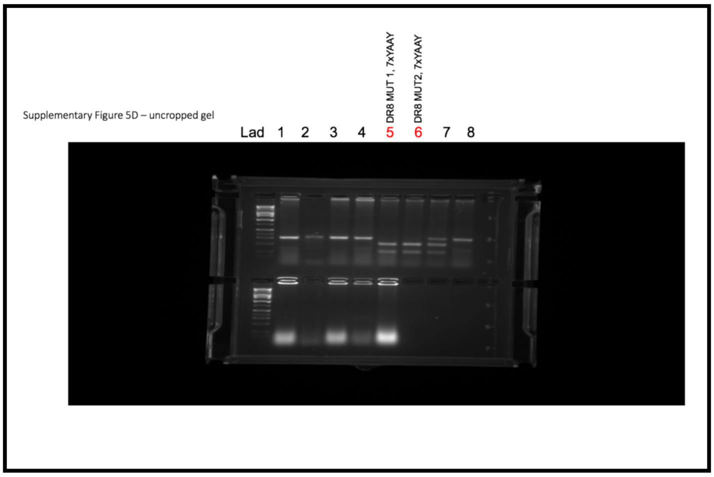

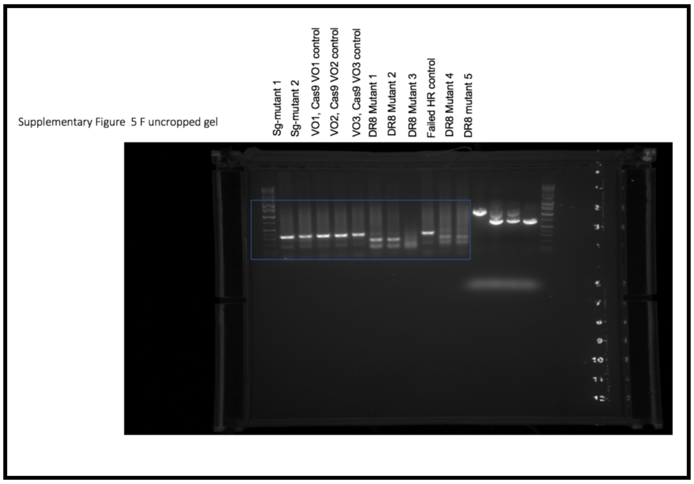

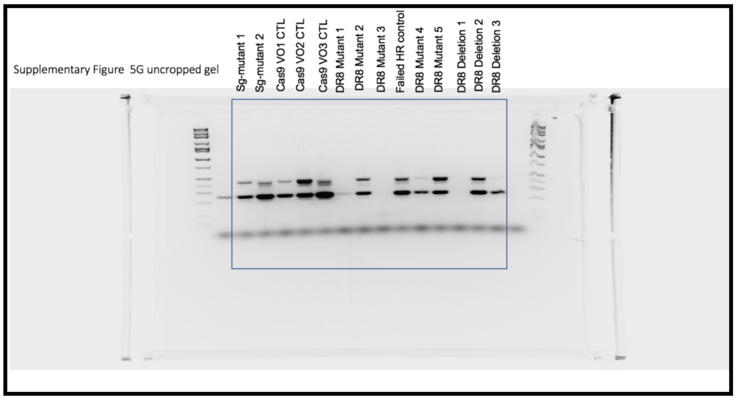

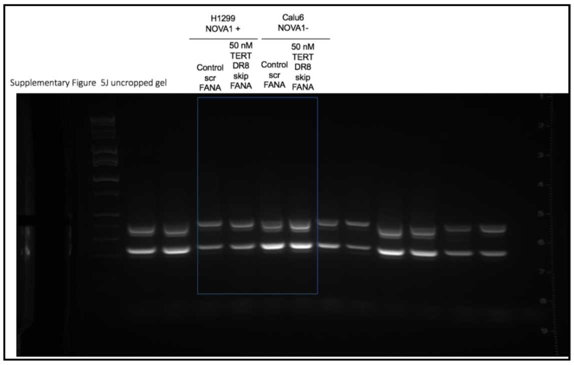

Supplementary References

1. Wong MS, Chen L, Foster C, Kainthla R, Shay JW, Wright WE. Regulation of telomerase alternative splicing: a target for chemotherapy. *Cell Rep* **3**, 1028-1035 (2013).

2. Yi X, Shay JW, Wright WE. Quantitation of telomerase components and hTERT mRNA splicing patterns in immortal human cells. *Nucleic Acids Res* **29**, 4818-4825 (2001).

3. Listerman I, Sun J, Gazzaniga FS, Lukas JL, Blackburn EH. The major reverse transcriptase-incompetent splice variant of the human telomerase protein inhibits telomerase activity but protects from apoptosis. *Cancer Res* **73**, 2817-2828 (2013).

4. Yi X, White DM, Aisner DL, Baur JA, Wright WE, Shay JW. An alternate splicing variant of the human telomerase catalytic subunit inhibits telomerase activity. *Neoplasia* **2**, 433-440 (2000).

5. Zhu S, Rousseau P, Lauzon C, Gandin V, Topisirovic I, Autexier C. Inactive C-terminal telomerase reverse transcriptase insertion splicing variants are dominant-negative inhibitors of telomerase. *Biochimie* **101**, 93-103 (2014).

6. Withers JB, Ashvetiya T, Beemon KL. Exclusion of exon 2 is a common mRNA splice variant of primate telomerase reverse transcriptases. *PLoS One* **7**, e48016 (2012).
