# Supplementary material for: NOVA1 regulates hTERT splicing and cell growth in non-small cell lung cancer
Source: Nat Commun. 2018 Aug 6;9:3112. doi: 10.1038/s41467-018-05582-x (PMC6079032; doi:10.1038/s41467-018-05582-x)
Supplement: Supplementary file 3 — Description of Additional Supplementary Files [file 41467_2018_5582_MOESM3_ESM.docx]

**Description of Additional Supplementary Files**

File Name: Supplementary Data 1

Description: **Tab 1.** Splicing factor siRNA sequences used in the minigene screen. **Tab 2.** Averaged minigene screen luciferase data from each target. A bar graph showing the 10 genes that overlapped between the screen and bioinformatics approaches. **Tab 3.** Gene identifiers of 110 genes that changed hTERT minigene splicing by 2-fold or greater in either direction (increase full length or increase minus beta hTERT). **Tab 4.** Gene identifiers of 93 genes that changed hTERT minigene splicing by 2-fold increase minus beta hTERT. **Tab 5.** Gene identifiers of 17 genes that changed hTERT minigene splicing by 2-fold increase in full-length hTERT. **Tab 6.** Gene identifiers of 12 genes correlated with endogenous hTERT full-length splicing in non-small cell lung cancer. **Tab 7.** Correlation of NOVA1 expression (mRNA, log2 transformed) and percent hTERT potential full-length. **Tab 8.** Pearson’s r regression analysis of the relationship between NOVA1 and hTERT. **Tab 9.** Correlation of NOVA1 expression (protein, log2 transformed) and percent hTERT potential full-length. **Tab 10.** Pearson’s r regression analysis of the relationship between NOVA1 and hTERT. **Tab 11.** Overlap analysis of genes in common between minigene screen and ‘discovery’ analysis of microarray data. **Tab 12.** Gene identifiers of 22 genes differentially expressed between high full-length TERT non-small cell lung cancer lines and low full-length TERT non-small cell lung cancer lines. The ‘network’ analysis. **Tab 13.** Overlap analysis of genes in common between minigene screen and ‘network’ analysis. **Tab 14.** Overlap analysis of genes in common between ‘network’ analysis and ‘discovery’ analysis.
